# Perioperative management of a patient with comorbid immune thrombocytopenia and antiphospholipid syndrome undergoing minimally invasive mitral valve surgery

**DOI:** 10.1186/s13019-026-04334-z

**Published:** 2026-06-04

**Authors:** Kazuki Mori, Hidenori Sako, Kenya Kizu, Takashi Shuto, Yuko Ogata, Shinji Miyamoto

**Affiliations:** 1 Department of Cardiovascular Surgery, Oita University, 1-1 Idaigaoka, Hasama, Yufu, 879-5593 Oita Japan; 2 Department of Cardiac Surgery, NHO Beppu Medical Center, Oita, Japan; 3 Department of Cardiovascular Surgery, Oita Oka Hospital, Oita, Japan; 4 Department of Hematology, NHO Beppu Medical Center, Oita, Japan

**Keywords:** Antiphospholipid syndrome, Immune thrombocytopenia, Immunoglobulin therapy, Minimally invasive cardiac surgery

## Abstract

**Background:**

Although rare, immune thrombocytopenia can coexist with antiphospholipid syndrome, creating a management dilemma between maintaining hemostasis and preventing thromboembolism. Herein, we report our strategic perioperative management of a patient with this complex hematological profile who underwent minimally invasive cardiac surgery.

**Case presentation:**

A 55-year-old man with chronic atrial fibrillation and severe mitral regurgitation was diagnosed with immune thrombocytopenia and antiphospholipid syndrome. A minimally invasive cardiac surgery approach was chosen to minimize surgical trauma and the risk of perioperative bleeding. The mitral valve exhibited thickened chordae and leaflet retraction accompanied by verrucous excrescences, which are highly suggestive of Libman–Sacks endocarditis. Despite attempts at valve repair using annuloplasty, artificial chordae, and cleft closure, significant mitral regurgitation persisted because of extensive fibrotic changes. Consequently, the procedure was converted to mitral valve replacement with a mechanical prosthesis. Although the patient’s platelet count immediately dropped to 12,000/µL postoperatively, prompt treatment with platelet transfusion and high-dose immunoglobulin therapy ensured rapid recovery. The patient was discharged without any complications, even after initiating anticoagulation therapy with warfarin.

**Conclusion:**

Managing cardiac surgery in patients with comorbid immune thrombocytopenia and antiphospholipid syndrome presents a significant therapeutic challenge. Based on our experience, a minimally invasive approach combined with a strategic perioperative protocol (specifically, postponing immunoglobulin therapy until the immediate postoperative period to facilitate rapid platelet recovery alongside anticoagulation) can achieve successful outcomes while balancing the conflicting risks of thrombosis and hemorrhage.

## Background

In patients with underlying hematological disorders, cardiac surgery requires meticulous attention to the high risk of perioperative bleeding and thromboembolic complications. We encountered a rare and complex case of mitral regurgitation (MR) complicated by immune thrombocytopenia (ITP) and antiphospholipid syndrome (APS), which was managed through a minimally invasive cardiac surgery (MICS) approach. The coexistence of ITP and APS presents a clinical paradox, balancing severe thrombocytopenia against a systemic prothrombotic state. In this report, we discuss the strategic perioperative management of these conflicting hematological conditions during cardiac intervention.

## Case presentation

A 55-year-old man who had been followed for atrial fibrillation since his 30s had a five-year history of MR. He began experiencing heart failure symptoms one year prior to admission. Echocardiography revealed left atrial enlargement associated with chronic atrial fibrillation. Severe MR was observed, resulting from anterior leaflet prolapse with intact chordae tendineae. The tricuspid valve showed moderate regurgitation because of tricuspid annular dilatation.

The patient’s initial laboratory tests revealed a significantly low platelet count of 19,000/µL with the presence of large platelets on the peripheral blood smear. The platelet-associated immunoglobulin G level was markedly elevated at 2150 ng/10^7^ cells. Bone marrow examination showed mildly increased megakaryocytes, but no findings suggestive of platelet production were observed. Furthermore, the patient tested positive for anticardiolipin β2-glycoprotein I antibodies, lupus anticoagulant, and anticardiolipin immunoglobulin G antibodies, leading to a diagnosis of APS. Laboratory tests were negative for anti-double-stranded DNA antibodies. The patient had no history of hemorrhagic or thromboembolic events. During the follow-up period, the platelet count gradually increased to 92,000/µL. Therefore, pharmacological intervention for hematological disorders was deferred, and anticoagulation therapy for atrial fibrillation was continued.

Preoperative laboratory tests revealed a platelet count of 90,000/µL. Since the patient was not anemic, 400 mL of autologous blood was stored for intraoperative use. Preoperative intravenous immunoglobulin (IVIG) therapy aimed at increasing the platelet count was withheld because of concerns regarding the increased risk of thrombosis associated with APS. Hence, we opted for a MICS approach for the valve procedure to minimize perioperative blood loss.

Cardiopulmonary bypass (CPB) was established via cannulation of the right internal jugular vein, right femoral vein, and right femoral artery. The surgical field was established through a 4-cm right fourth intercostal mini-thoracotomy. A 10-mm camera port was inserted in the fourth intercostal space at the midaxillary line, with additional 5-mm ports placed in the third and sixth intercostal spaces. A small incision was made in the fifth intercostal space along the posterior axillary line for the insertion of a Chitwood clamp. A cardioplegia cannula was inserted into the ascending aorta. After the patient was cooled to 30 °C, the aorta was cross-clamped, and cardioplegic arrest was achieved with antegrade cardioplegia. After left atriotomy, the left atrial appendage ostium was closed from within the left atrium using a running suture. We performed para-annular left atrial plication using 2 − 0 polyester sutures.

**Fig. 1:**
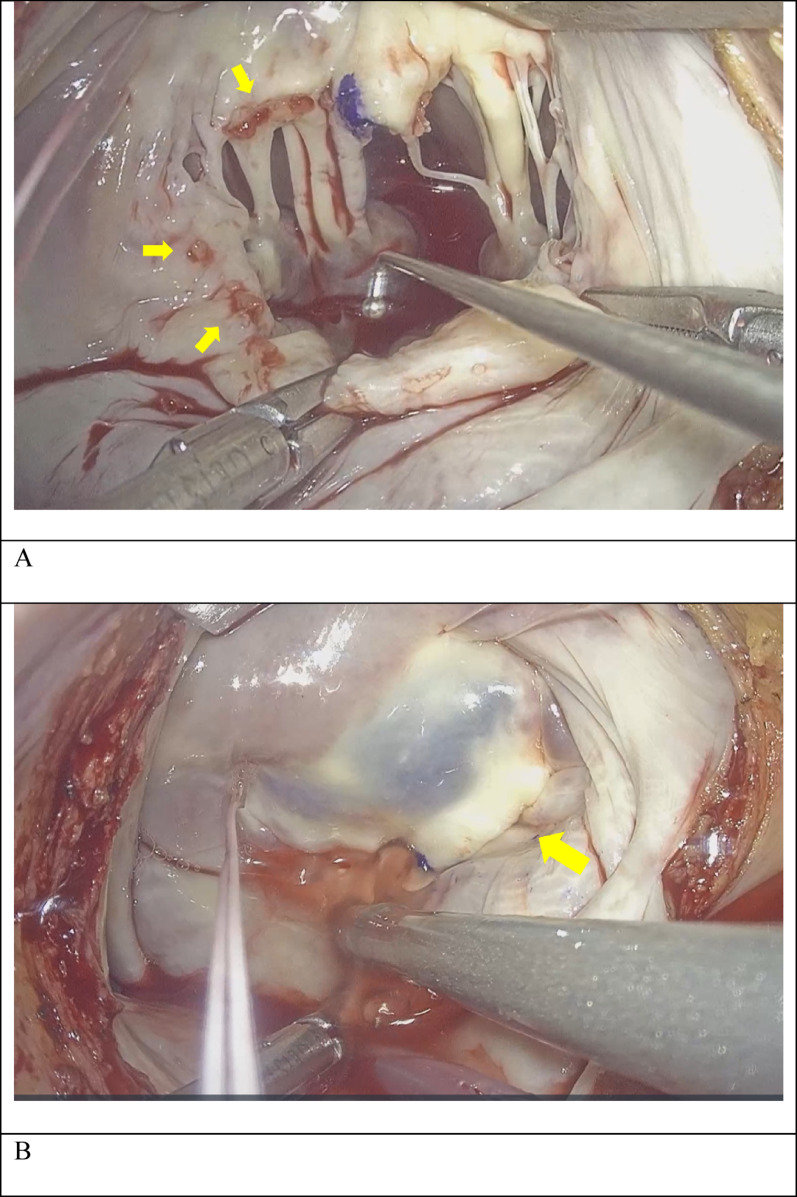
Intraoperative findings of the mitral valve. (**A**) The mitral valve showed significant leaflet retraction between the A1 segment and lateral A2 segment, secondary to thickened chordae originating from the anterior papillary muscle. Verrucous excrescences (arrows) are visible along the thickened leaflet edges, consistent with the typical presentation of Libman–Sacks endocarditis in antiphospholipid syndrome. (**B**) Coaptation was insufficient at the lateral side, whereas the medial side showed relative prolapse (arrow)

The mitral valve exhibited leaflet retraction between the A1 segment and the lateral A2 segment, associated with thickened chordae originating from the anterior papillary muscle. Accordingly, the medial side of the anterior leaflet exhibited relative prolapse. We observed verrucous excrescences suspicious of vegetations along the thickened leaflet tissue (Fig. 1). These findings strongly supported the diagnosis of Libman–Sacks endocarditis associated with APS. After implanting a 32-mm Physio II Ring (Edwards Lifesciences, Irvine, CA, USA), we addressed the residual prolapse at the medial A2 segment by anchoring artificial chordae to correct the regurgitation. Despite a minor leak during the saline test, left atrial closure was performed because the degree of regurgitation was less than the initial assessment. Subsequently, we performed a right atriotomy and secured a 32-mm Physio Tricuspid Ring (Edwards Lifesciences, Irvine, CA, USA) to the tricuspid annulus.

After the aorta was declamped and spontaneous heartbeat returned, transesophageal echocardiography revealed residual moderate MR. Consequently, we reinstituted cardioplegic arrest to inspect the valve and closed the cleft at the A2 segment using 5 − 0 polypropylene sutures. Intraoperative transesophageal echocardiography showed no significant improvement in MR severity following the second declamping. We determined that further attempts at valve repair were unfeasible as continued intervention would further prolong the CPB time and exacerbate the risk of perioperative bleeding. Thus, we proceeded with mitral valve replacement using a 29-mm St. Jude Medical mechanical valve (St. Jude Medical, St. Paul, MN, USA). Because of these repeated interventions, the CPB time was prolonged to 345 min.

**Fig. 2:**
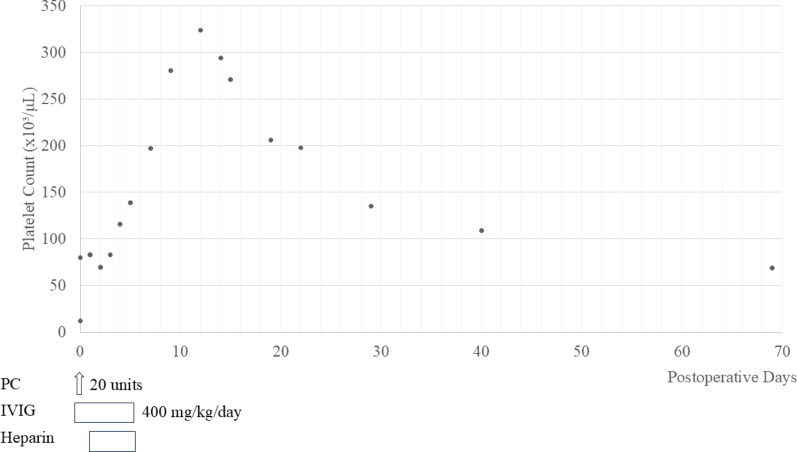
Perioperative changes in the platelet count. The preoperative platelet count was 90,000/µL, which immediately decreased to 12,000/µL after cardiopulmonary bypass. However, the platelet count recovered to 83,000/µL on the first postoperative day following the administration of high-dose IVIG and a transfusion of 20 units of PC. The count peaked at 324,000/µL on postoperative day 12, subsequently returning to the preoperative baseline by postoperative day 70. PC, platelet concentrate; IVIG, intravenous immunoglobulin

The patient’s platelet count immediately decreased to 12,000/µL after surgery. To prevent postoperative bleeding, 20 units of platelet concentrate were transfused following the administration of high-dose IVIG. The IVIG therapy was continued at a dose of 20 g/day (400 mg/kg/day) for five consecutive days. The patient showed no signs of excessive drain hemorrhage. The platelet count increased to 83,000/µL the next day and continued to increase thereafter (Fig. 2). The patient was extubated on the first postoperative day and required two days of management in the intensive care unit. Postoperative anticoagulation, comprising continuous heparin from day 1 and warfarin from day 2, was carefully managed to prevent valve thrombosis. The patient was discharged on postoperative day 15 without any complications, including hemorrhage or thromboembolism. At the outpatient follow-up, the platelet count had returned to preoperative levels, and no significant further decline was observed. The patient’s anticoagulation therapy is strictly managed with a target PT-INR of 2.5–3.5, following guidelines for mechanical valves.

### Discussion and conclusion

The perioperative management of patients undergoing cardiac surgery requires a delicate balance between preventing thrombosis and controlling bleeding. However, the presence of comorbid ITP and APS further exacerbates this challenge as clinicians must navigate the opposing risks of severe thrombocytopenia and hypercoagulability. According to previous studies, 16%–37.8% of patients with ITP test positive for antiphospholipid antibodies [1–3]. The presence of lupus anticoagulant is associated with a significantly increased risk of thrombotic events, even with a low platelet count [1, 3]. Furthermore, thrombocytopenia is frequently observed in patients with APS, with mechanisms involving platelet consumption because of chronic activation, the presence of antiplatelet autoantibodies, and microthrombus formation [4].

Cardiovascular surgery involving CPB inevitably leads to a decreased platelet count. Therefore, perioperative platelet management is particularly important in patients with ITP. The standard treatment for ITP includes corticosteroids, IVIG, thrombopoietin receptor agonists, splenectomy, and rituximab [4, 5]. Although corticosteroids and immunosuppressive therapies are established options, they were intentionally withheld because their efficacy can be inconsistent depending on the patient. Furthermore, we prioritized avoiding the increased risk of perioperative infections, including surgical site infections, which are a significant concern during cardiac surgery. Preoperative intervention was also deferred because the baseline platelet count of 90,000/µL was deemed adequate, given that the MICS technique effectively minimizes perioperative hemorrhage compared to conventional approaches.

We relied on IVIG to achieve the necessary perioperative increase in platelets. However, we carefully managed the preoperative phase, considering that aggressive platelet augmentation could be hazardous for a patient with APS because of the increased risk of thrombosis. IVIG increases the platelet count within 12–24 h; however, this effect is temporary and typically diminishes after about a month [4]. IVIG administration after CPB prevents the inadvertent loss of the agent caused by systemic hemodilution and adherence to the synthetic surfaces of the bypass circuit. This ensures that the full dose is available to stabilize the platelet count during the most vulnerable hemostatic period. Postoperatively, the patient’s platelet count showed a rapid upward trend, reaching 324,000/µL on postoperative day 12. On the other hand, such a precipitous increase in the platelet count in a patient with APS and a mechanical valve represents a critical window of significantly heightened thrombotic risk. To mitigate this risk, we initiated continuous heparin infusion on postoperative day 1 immediately after confirming the absence of drain discharge and the start of the platelet recovery. The dosage was carefully titrated to maintain an activated partial thromboplastin time of at least twice the baseline. Simultaneously, warfarin was started on postoperative day 1. Through these strategic measures, we successfully averted both perioperative hemorrhagic and thrombotic events.

Although the valve repair was ultimately unsuccessful, the surgical view provided by the MICS approach was excellent, and the failure was attributable to the complex valve morphology rather than the surgical approach itself. While the extended CPB time contributed to the profound postoperative thrombocytopenia, the MICS technique remained instrumental in the patient’s favorable outcome. The reduced tissue dissection and smaller wound area effectively mitigated the bleeding risk from surgical surfaces and bone marrow, which might have been uncontrollable with a conventional sternotomy [6]. Consequently, we achieved complete hemostasis and avoided postoperative hemorrhage despite the unexpected severity of the subsequent thrombocytopenia.

In the present case, mitral valve repair was unsuccessful, requiring valve replacement. The selection of a prosthetic valve in patients with comorbid ITP and APS remains a subject of debate [7]. Considering the conflicting risks of bleeding because of ITP and the prothrombotic state of APS, avoiding mechanical valves may be preferable to eliminate the need for lifelong anticoagulation and minimize the risk of prosthetic valve thrombosis. However, the necessity for future reoperation would subject the patient to the same complex perioperative challenges considering the limited durability of bioprosthetic valves. Furthermore, accelerated structural valve deterioration of bioprosthetic valves has been reported in patients with Libman–Sacks endocarditis [8]. We opted for a mechanical valve based on the following pragmatic considerations. First, the patient’s age of 55 years aligns with the general recommendation for mechanical valves to ensure long-term durability. Second, in a patient with such a complex hematological profile, structural valve deterioration would necessitate a re-operation, re-exposing the patient to an extremely hazardous perioperative management environment characterized by both life-threatening hemorrhage and thrombosis. Therefore, we concluded that selecting a mechanical valve was the most reasonable and sustainable choice, as it avoids the high-risk scenario of future reoperation while aligning with the necessary long-term management of APS.

Based on our experience, the simultaneous administration of high-dose IVIG and platelet transfusion upon weaning from CPB could be considered as a viable management strategy to ensure immediate hemostatic recovery for patients with ITP undergoing cardiovascular surgery involving CPB. This proactive approach facilitates a rapid increase in platelet count and promotes stable postoperative hemostasis.

## Data Availability

No datasets were generated or analysed during the current study.

## Abbreviations

MR: Mitral regurgitation
ITP: Immune thrombocytopenia
APS: Antiphospholipid syndrome
MICS: Minimally invasive cardiac surgery
IVIG: Intravenous immunoglobulin
CPB: Cardiopulmonary bypass

## References

[1] Funauchi M, Hamada K, Enomoto H, Ikoma S, Ohno M, Kinoshita K, et al. Characteristics of the clinical findings in patients with idiopathic thrombocytopenic purpura who are positive for anti-phospholipid antibodies. Intern Med. 1997;36:882–5. 9475243 10.2169/internalmedicine.36.882

[2] Fayard D, Lobbes H, Pereira B, Ruivard M. Risk of thrombosis associated with antiphospholipid antibodies in immune thrombocytopenic purpura: a single center retrospective study of 152 patients. Thromb Res. 2023;221:7–9. 36435049 10.1016/j.thromres.2022.11.008

[3] Diz-Küçükkaya R, Hacihanefioğlu A, Yenerel M, Turgut M, Keskin H, Nalçaci M, et al. Antiphospholipid antibodies and antiphospholipid syndrome in patients presenting with immune thrombocytopenic purpura: a prospective cohort study. Blood. 2001;98:1760–4. 11535509 10.1182/blood.v98.6.1760

[4] Bashyal KP, Shah S, Ghimire C, Balmuri S, Chaudhary P, Karki S, et al. Primary immune thrombocytopenic purpura (ITP) and ITP associated with systemic lupus erythematosus: a review of clinical characteristics and treatment modalities. Int J Rheumatol. 2024;2024:6650921. 38464849 10.1155/2024/6650921 PMC10923624

[5] Sun S, Urbanus RT, Ten Cate H, de Groot PG, de Laat B, Heemskerk JWM, et al. Platelet activation mechanisms and consequences of immune thrombocytopenia. Cells. 2021;10:3386. 34943895 10.3390/cells10123386 PMC8699996

[6] Pojar M, Karalko M, Dergel M, Vojacek J. Minimally invasive or sternotomy approach in mitral valve surgery: a propensity-matched comparison. J Cardiothorac Surg. 2021;16:228. 34376231 10.1186/s13019-021-01578-9 PMC8353832

[7] Bouma W, Klinkenberg TJ, van der Horst IC, Wijdh-den Hamer IJ, Erasmus ME, Bijl M, et al. Mitral valve surgery for mitral regurgitation caused by Libman-Sacks endocarditis: a report of four cases and a systematic review of the literature. J Cardiothorac Surg. 2010;5:13. 20331896 10.1186/1749-8090-5-13 PMC2859362

[8] Chalvon NB, Costedoat-Chalumeau N, Pennaforte JL, Servettaz A, Boulagnon Rombi C, Gavand PE, et al. Severe Libman-Sacks endocarditis complicating antiphospholipid syndrome: a retrospective analysis of 23 operated cases. Rheumatology (Oxford). 2023;62:707–15. 35686908 10.1093/rheumatology/keac315

